# The Medusae Fossae Formation as the single largest source of dust on Mars

**DOI:** 10.1038/s41467-018-05291-5

**Published:** 2018-07-20

**Authors:** Lujendra Ojha, Kevin Lewis, Suniti Karunatillake, Mariek Schmidt

**Affiliations:** 1 Department of Earth and Planetary Sciences, Johns Hopkins University, Baltimore, MD 21218 USA; 2 Department of Geology and Geophysics, Louisiana State University, Baton Rouge, LA 70803 USA; 3 Department of Earth Sciences, Brock University, St. Catharines, ON L2S 3A1 Canada

## Abstract

Transport of fine-grained dust is one of the most widespread sedimentary processes occurring on Mars today. In the present climate, eolian abrasion and deflation of rocks are likely the most pervasive and active dust-forming mechanism. Martian dust is globally enriched in S and Cl and has a distinct mean S:Cl ratio. Here we identify a potential source region for Martian dust based on analysis of elemental abundance data. We show that a large sedimentary unit called the Medusae Fossae Formation (MFF) has the highest abundance of S and Cl, and provides the best chemical match to surface measurements of Martian dust. Based on volume estimates of the eroded materials from the MFF, along with the enrichment of elemental S and Cl, and overall geochemical similarity, we propose that long-term deflation of the MFF has significantly contributed to the global Martian dust reservoir.

## Abstract (web summary)

Martian dust is globally enriched in S and Cl and has a distinct mean S:Cl ratio. Here the authors identify that the largest potential source region for Martian dust based on analysis of elemental abundance data may be the Medusae Fossae Formation.

## Introduction

Dust is ubiquitous on Mars and plays a key role in contemporary atmospheric and surface processes. Dust in the Martian atmosphere absorbs and scatters solar and infrared radiation, and plays a dominant role in controlling the Martian atmospheric temperature^1,2^. Atmospheric dust loading can cause significant atmospheric shielding, decreasing the daytime near-surface temperature by tens of Kelvins while increasing the temperature in the middle atmosphere by many tens of Kelvins^3^. Positive feedbacks associated with dust-lifting into the Martian atmosphere can also lead to regional and planet-encircling global dust storms. Atmospheric dust is a major concern for robotic and future human exploration; the settling of dust on the solar panels of landed spacecraft significantly decreases their power output over time^4^, and dust contamination can impact spacecraft instruments and hardware^5^, and create health hazards^6^. Globally, it is estimated that 2.9 × 10^12^ kg/yr of dust is exchanged between the surface and atmosphere of Mars^7^, making dust activity one of the most dynamic and prevalent contemporary geological processes on the planet.

The physical and chemical characteristics of dust provide information on the geological processes responsible for their formation. Early terrestrial work suggested glacial grinding to be the only natural process that efficiently converted sand-sized (0.06–2 mm diameter) quartz particles into a silt-sized (<62.5 µm) product (e.g. ref.^8^). In more recent years, a number of other mechanisms, such as frost shattering, fluvial comminution, volcanism, eolian abrasion, and salt weathering, have been proposed to account for dust deposits that occur near arid or semi-arid regions where glaciers have not existed^9–12^. The relative effectiveness of these various silt-producing mechanisms has been investigated in laboratory settings^10^. The results indicate that fluvial and eolian activity are most effective at producing silt-sized particles over short periods of time, while glacial grinding is found to be an inefficient silt-producing mechanism over short time scales^10^. Over the course of more than four billion years of Martian geological history, the relatively short lifetime of wet-based glacial activity (e.g. refs.^13,14^), likely played a minor role in dust formation. Fluvial comminution in a high energy, mixed sediment environment can reduce sand-sized particles to dust by a sequence of mechanical processes^10^, but the relatively short lifetime of fluvial features on Mars (e.g. ref.^14^), also suggest that this mode of dust formation likely did not contribute significantly to the present day dust reservoir of Mars. The particle size distribution of Martian soil observed by microsocopy at the Phoenix landing site suggests the Martian dust to be predominantly the product of eolian weathering under extremely dry conditions^15^. The presence of olivine in the Martian dust, a mineral highly susceptible to alteration in liquid water, provides additional evidence that liquid water did not play a major role in its formation^16^. Frost shattering and salt weathering are two of the most inefficient modes of dust formation^10^, and therefore we do not consider them to be a major source of Martian dust.

The abundance of volcanoes on Mars, and particularly indicators of explosive volcanism^17,18^ suggest that ancient ash deposits could be a significant contributor to the Martian dust reservoir. As a primary pathway, this mode of dust formation would likely be most dominant during the early history of Mars when volcanism was most active^13,19^. In comparison to volcanism, impact cratering is known to be an ongoing process on Mars (e.g. ref.^20^). Fragmentation of local rocks from impacts has been suggested to be the main source of sands on Mars, but fragmentation theory and measurements from nuclear explosion craters indicate that impacts do not produce significant quantities of dust-sized materials directly^21,22^.

In the absence of stable liquid water and active volcanism, the most active and efficient geological process responsible for dust-formation on Mars today is likely abrasion of mechanically weak rock units, including widespread sedimentary deposits, and eolian breakdown of saltating sand-sized particles^23^. The abundance of wind-carved ventifacts on Mars demonstrates that eolian abrasion has been one of the most significant recent erosional processes on the planet^24–30^, while in situ observations of the active Bagnold Dune in Gale crater indicate that saltation is sustainable even under the current low-density atmosphere^31^. The rate of abrasion depends on the composition of the target and abrading particles, but in areas of active sand migration, abrasion rates between 1 and 50 μm/yr have been estimated^32^. Laboratory experiments suggest a higher rate of abrasion on Mars than on Earth due to the lower atmospheric pressure and subsequent higher saltation friction speeds^33,34^. Further, these experiments have shown that abrasion is most efficient on fine-grained rocks of intermediate hardness^33^. Evidence of large-scale removal of eroded material by deflation can be seen in certain areas of Mars as decimeter-scale features, such as moats, wind tails, and lag deposits. Near the equatorial region of Mars, huge yardangs up to 10s of kilometers in scales can be observed (e.g. ref.^35^), highlighting the considerable role eolian forces have played on the surface erosion on Mars. Eolian dust formation is also possible via the inter-particle collision of coarser grains, which can produce dust by complete fracture, or via removal of sharp corners from parent materials^10,36^. For these reasons, we favor both the creation and dispersion of dust on Mars in the post-Noachian [3.7 billion years–present day] climate to be primarily grounded in eolian processes.

Based on orbital and in situ investigations, Martian dust is known to be consistently and markedly enriched in both sulfur (S) and chlorine (Cl) relative to the average Martian soil^37–39^. Data from the Mars Exploration Rovers (MER) showed that bright dust deposits on opposite sides of the planets had similar compositions, suggesting that the Martian dust comprises a global unit, and is not strongly influenced by the composition of local rocks^37^. Here we show that a large sedimentary unit called the Medusae Fossae Formation (MFF) has the highest abundance of S and Cl, and provides the best chemical match to surface measurements of Martian dust. Based on volume estimates of the eroded materials from the MFF, along with the enrichment of elemental S and Cl, and overall geochemical similarity, we propose that long-term deflation of the MFF has significantly contributed to the global Martian dust reservoir.

## Results

### Chemical analyses

Based on orbital and in situ investigations, Martian dust is known to be composed of framework silicates, mostly feldspar, with minor amounts of olivine, pyroxene, amorphous phases, and magnetite^16,40^, distinct from terrestrial dust which is dominated by a more quartz-rich granitic lithology. In addition to these crystalline phases, in situ investigations from past and current rovers have revealed the dust to be consistently and markedly enriched in both S and Cl relative to the average Martian soil^37–39^. Nanophase ferric oxide (npOx) abundances determined by the MER Mössbauer spectrometer correlate with SO_3_ abundances in soils in Gusev crater^37^, and are thought to be an important constituent of the global dust. In situ APXS data from the Mars Science Laboratory (MSL) has further corroborated the model of a globally homogenous dust reservoir with a consistent molar S:Cl ratio of 3.7 ± 0.7^39^.

The geochemistry of the Martian dust observed in situ by landed spacecraft can be compared with the shallow subsurface composition of the Martian crust as determined with the Mars Odyssey Gamma Ray Spectrometer Suite (GRS) observations. GRS mapping provides elemental geochemistry of the near-surface regolith of Mars, by measuring the spectrum of gamma photons emitted from the Martian surface; characteristic spectral peaks from specific nuclear reactions allow the quantification of most major rock-forming elements, along with select minor and trace elements (Al, Ca, Cl, Fe, H, K, S, Si, Th)^41^. Peak area above continuum can be used to infer mass fraction (wt%) of each element over an area of the planet’s surface, leading to chemical abundance maps. New GRS mapping results include sulfur abundance at the decimeter-depth scale throughout the mid-to-low latitudes of Mars.

**Fig. 1:**
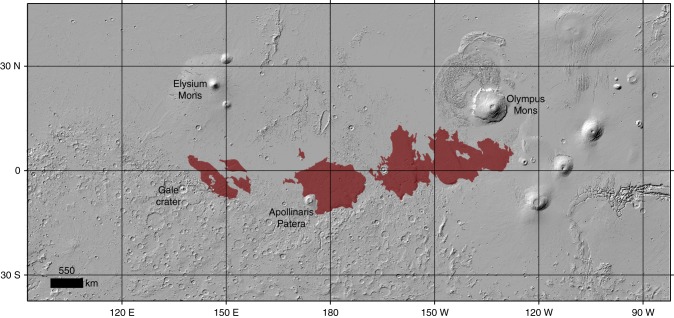
The geographical extent of the MFF in dark brown on top of a MOLA shaded relief. The volcanos Elysium Mons, Olympus Mons, and Apollinaris Patera, along with Gale crater are annotated to provide geographical context

**Fig. 2:**
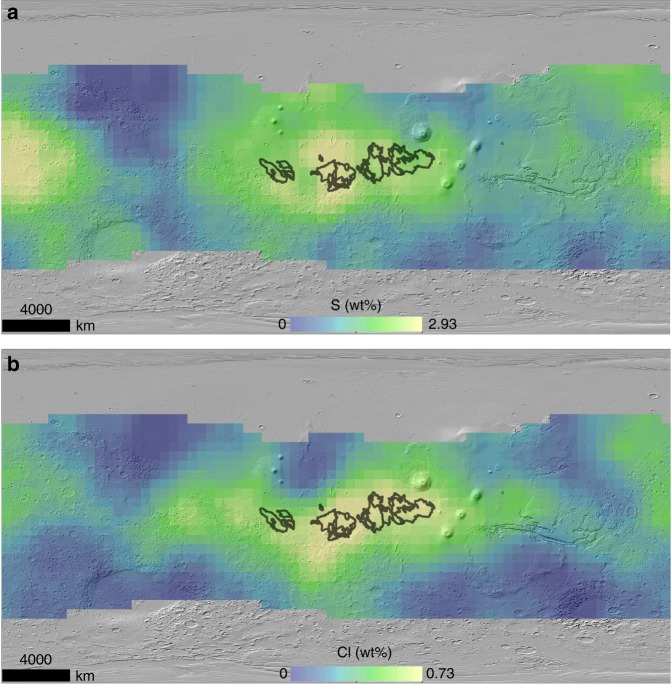
GRS-derived abundance maps for S and Cl. GRS-derived elemental abundance maps for S (**a**) and Cl (**b**) in wt%, overlain on a MOLA shaded relief. The outline of the MFF is shown in black

**Fig. 3:**
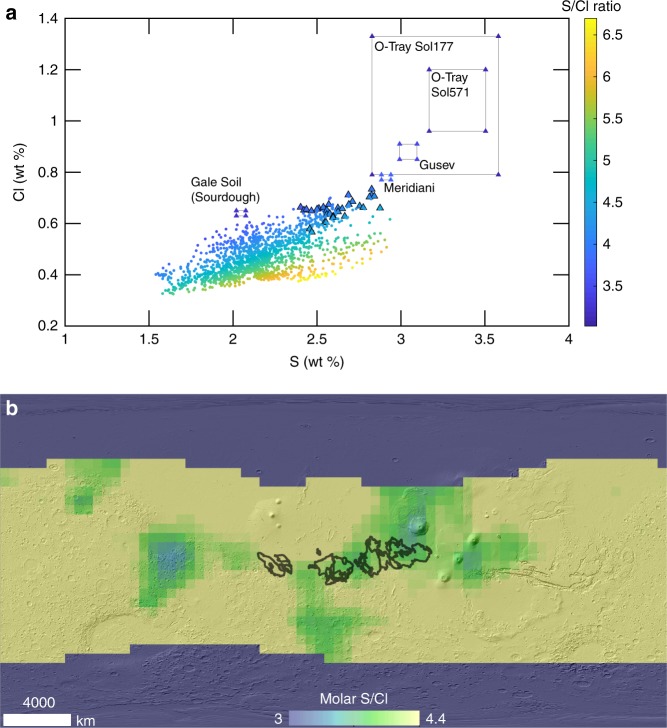
S:Cl molar ratio of the MFF and other areas on Mars. **a** S:Cl molar ratio as a function of S and Cl for each pixel within the GRS-mapped regions of Mars. The points with triangle outlines correspond to the GRS observations that fall within the MFF. The rectangular boxes represent the S and Cl abundances of the Martian dust observed by the MER rovers^38,52^ and MSL^39^. **b** Map showing regions on Mars with S:Cl ratio between 3 and 4.4

The highest enrichment of S and Cl on Mars is found within the massive yardang fields of the MFF and surrounding areas (Figs. 1 and 2). The MFF lies in the equatorial region of Mars near the topographic dichotomy separating the northern lowlands from the southern highlands of the planet (Fig. 1). The MFF has a relatively young surface exposure age and was likely emplaced during the Hesperian era^42,43^ [3.7–3.0 billion years]. The depositional history of the MFF is not entirely understood, though several hypotheses have been proposed, including glacial activity emplacing paleo-polar deposits which incorporated eolian sediment^44^, direct atmospheric deposition of suspended eolian sediment^45^, coarser-grained ignimbrite deposits from pyroclastic density currents^46^, and fine-grained distal ashfall from volcanic eruptions^45,47–50^. From image and topographic mapping, the current areal extent of the MFF is estimated to be 2 × 10^6^ km^2^ (20% of the continental United States) but could have once covered an area greater than 5 × 10^6^ km^2^ (50% of the continental United States)^48^. Despite the huge volume of material eroded from the MFF^48^, there are relatively few depositional bedforms around the MFF, suggesting that most of the materials being eroded are fine-grained enough to be suspended in the atmosphere and transported long distances^45^ (<10s μm). We observe a range of 2.40–2.88 wt% for S and 0.57–0.74 wt% for Cl from all the GRS pixels that lie within the geological boundary of the MFF (Fig. 3). From these observations and uncertainty associated with the GRS measurements, we estimate a mean S and Cl wt% of 2.62 ± 0.24 and 0.65 ± 0.04, respectively. These values may be compared to the mid-latitudinal average of 2.17 ± 0.23 wt% S and 0.47 ± 0.03 wt% Cl, representing a mean enrichment factor within the MFF of 1.2× and 1.4×, respectively. Combining the range of S and Cl estimate of the MFF, we calculate an average molar S:Cl ratio of 4.1, which falls within the range of S:Cl ratios observed for the Martian dust^39^ (3.0–4.4) (Fig. 3). The MFF is not the only region on Mars with an S:Cl ratio within this range (Fig. 3b, Supplementary Fig. 1), however, the MFF and nearby areas are the only region on Mars with significant enrichment of both S and Cl, in combination with an S:Cl molar ratio that is consistent with the Martian dust (Fig. 3, Supplementary Fig. 1). The statistical significance of S and Cl enrichment at the MFF was evaluated using an enhanced Student’s *t*-test parameter that weights for measurement error^51^ (see the Methods section). The results from this test suggest that the MFF is the only region on Mars with a significant enrichment of both S and Cl relative to the rest of Mars mapped by GRS (directional deviation significant at 94–98% confidence) (Supplementary Fig. 2).

The alpha particle X-ray spectrometers (APXS) on MER and MSL also provide abundances of many other elements in the Martian dust^37–39,52^. As seen in situ, no other elements in the Martian dust exhibit a signature as distinct as that of S and Cl relative to other local rocks and soils^39^ (Supplementary Figs. 3 and 4). We may, however, still compare their measured abundance to GRS-derived values to assess the geochemical similarity between the bulk regolith of Mars and the mean bulk composition of the Martian dust (e.g. ref.^51^). To address possible systematic differences between the GRS and APXS data and to understand how volatile species, such as S and Cl vary across Mars relative to major elements such as Si, we used ratios of wt% instead of the wt% themselves. Chemical ratios are calculated using H_2_O-free normalized SiO_2_ and as expected, show the MFF to have a distinct trend of high Cl/SiO_2_ and SO_3_/SiO_2_, resembling the Martian dust (Supplementary Figs. 3 and 4). As with APXS measurements of the dust, no other elements that we examined from GRS data (K, Fe, Ca, and Al) exhibited any distinct trends in the MFF (Supplementary Figs. 3 and 4). We also compared the normalized elemental abundances of Si, Fe, Ca, K, S, and Cl in the Martian dust with the rest of the area of Mars mapped by GRS (Supplementary Fig. 5). The in situ elemental abundance data of the Martian dust and GRS data were normalized by dividing the wt% of each element by its average value in Martian soil as observed in situ^53^ and by the mean mid-latitudinal GRS values, respectively. Large portions of the planet may match the elemental chemistry of Martian dust in one or a few elements (Supplementary Fig. 5). However, the only region of Mars that falls within the range observed for Martian dust for each of Si, Fe, Ca, K, S, and Cl lies within the MFF and adjacent areas that may have contributions from the MFF (Supplementary Fig. 5). Among the extensive MFF deposits, the area that produces the best fit to the APXS-derived dust chemistry consists of several GRS pixels near Lucus Planum within the MFF and nearby surrounding areas (Supplementary Fig. 5).

In situ measurement of Martian dust at Rocknest by the ChemCam instrument onboard MSL indicates that Martian dust may also be hydrated at the surface^54^. The source of hydration in Martian dust could be due to the presence of adsorbed atmospheric water, an −OH or H_2_O bearing crystalline mineral, and/or amorphous hydrated phases. ChemCam did not detect significant variations in H concentration with surface humidity^54^, which would be expected if the major source of the hydration signature in the Martian dust was adsorbed atmospheric water. CheMin, also onboard MSL, found no evidence of hydrated crystalline minerals in the Rocknest soil at Gale, so the hydration of Martian dust most likely corresponds to a hydrated amorphous component. GRS-derived H_2_O abundances reveal a notable enrichment that overlaps spatially with the central MFF lobes near Lucus Planum (Supplementary Fig. 6). We estimate the mean H_2_O content of the MFF to be 5.19 ± 1.83 wt%, attributed most simply to chemically bound H_2_O in the MFF. Previously, H_2_O and S were found to be correlated in the Martian midlatitudes with trends more consistent with chemically bound H_2_O as opposed to adsorption^55^. To further assess the relationship between H and S at the MFF, we computed linear regression fits to the GRS data: wt%(H_2_O) = *m* × wt%(S) + *C*, where *m* represents the stoichiometric association between H_2_O and S (Supplementary Fig. 7). We find the H_2_O and S in the MFF to be highly correlated (correlation coefficient of 0.80), more strongly than in the midlatitudes of Mars as a whole (correlation coefficient of 0.66) (Supplementary Fig. 7). The corresponding negative intercept for the MFF (*C* = −12) compared to the bulk Mars (*C* = −1.7) is consistent with the lack of excess H_2_O in the bulk MFF. Instead, all H_2_O can be interpreted to be chemically bound to S in the MFF, reinforced by S modeling over 60% of the H_2_O variance (Supplementary Fig. 7). Although the application of Pixon-based statistical image reconstruction to neutron spectroscopy-derived hydrogen abundance has been used to suggest H_2_O abundance of 35–40 wt% in the MFF^56^, more validation is necessary before integrating these results with the separately derived GRS elemental data at the pixel scale.

The MFF contains the highest levels of Cl and S on Mars, though the GRS-estimated values are still lower than in situ APXS measurements of air-fall dust (Fig. 3). The apparent difference in the numerical abundance of Cl and S between the MFF and the Martian airfall dust can be attributed most simply to the differences in the spatial sampling scale^57^. Given the coarse resolution of GRS and the relatively small areal extent of the MFF, GRS pixels centered over the region can have a maximum of 77% of the cumulative gamma spectrum originating from within the geologically inferred extent of the MFF (Supplementary Fig. 8). Therefore, the relatively coarse resolution of GRS and limited contribution from the MFF to GRS field of view causes a spatial dilution effect, which will underestimate the actual elemental enrichment in the MFF^57,58^ (Supplementary Fig. 8). While the absolute abundance of S and Cl can vary among landing sites due to different sampling and measurement conditions, the relative enrichment of S and Cl compared to other elements is most distinctive of the Martian dust. Regardless, there are no other regions on Mars with the same level of both S and Cl enrichment as the MFF along with an S:Cl ratio that overlaps with the dust measured in situ (Supplementary Figs. 1 and 2). In contrast, a region near Meridiani Planum that shows significant enrichment of sulfur in the GRS data lacks a corresponding enrichment in chlorine (Fig. 2, Supplementary Fig. 2). Orbital infrared spectral detections suggest the likely source of S in this region to be sulfates, whereas no sulfates have been detected within the MFF or nearby regions (Supplementary Fig. 9).

Sulfur and chlorine are major components of typical volcanic gases on the Earth (e.g. ref.^59^) their enrichment in the MFF provides further support for a pyroclastic depositional origin of the unit (e.g. ref.^50^). The enrichment of sulfur in the MFF may help us understand the early evolution of the Martian atmosphere and hydrosphere, and serve as a proxy for SO_2_ and H_2_S emission over the time of its formation. The outgassing of sulfur from Martian volcanoes could have served as a viable alternative to CO_2_ for a sustained greenhouse effect (e.g. ref.^60^). In an atmosphere with significant quantities of reduced sulfur gases, SO_2_ would play a particularly important role not only climatically as a powerful greenhouse gas but also in the chemistry of the surface environment. High sulfate contents can dramatically lower the pH of surface waters suppressing carbonate saturation in favor of sulfate minerals, thereby explaining the lack of widespread carbonate minerals on the Martian surface^60^.

### Volumetric calculation of dust on Mars

Based on the volume of material estimated to have been eroded from the MFF, we can calculate the contribution to the present-day dust inventory of Mars. The areal extent of the MFF currently exceeds 2 × 10^6^ km^2^ but may have covered an area greater than 5 × 10^6^ km^2^^48^. The current mean thickness of the MFF exceeds 600 m^48^. A comparable mean thickness between 100 and 600 m over the eroded areas would imply an eroded volume exceeding 3 × 10^5^–1.8 × 10^6^ km^3^. If distributed globally, this eroded volume of the MFF would be equivalent to a 2–12 m global layer of dust. We can compare this eroded volume to some of the largest known reservoirs of dust on Mars. Totaling all the dust present in the north polar-layered deposit (NPLD), the south polar-layered deposit (SPLD), Arabia, Tharsis, Amazonis, Elysium, and other dusty regions, we estimate an equivalent ~3 m global layer of dust, a comparable volume of material as that estimated to have been removed from the MFF through erosion (see the Methods section). These volume estimates illustrate that the MFF would have made a major contribution to the present-day dust inventory of Mars. Although these other regions are inferred to contain significant quantities of dust, they do not exhibit the same levels of S and Cl enrichment seen in the MFF; we attribute this to alteration or mixing with local materials, as has been suggested from geochemical comparisons across the Amazonis, Tharsis, and Arabia regions^61^.

The compositional convergence of the MFF with the chemistry of dust analyzed by both the MER and MSL rovers suggests the role of the MFF as the single largest global dust source on Mars, continuing to the present day. Observed enrichment of both sulfur and chlorine, along with the observed S:Cl ratio, match defining characteristics of dust observed in situ from these landed missions. The fine-grained nature and morphologic features indicating extensive erosion, suggest that the MFF has contributed a volume of fines comparable to all other known dust reservoirs on the planet. This reverses prior interpretations of the MFF as a long-term dust-sink and resolves a major unknown in the source-to-sink pathways of the Martian dust cycle.

## Methods

### Chemical analyses

The chemical maps (of Al, Ca, Cl, Fe, K, S, Si, Th, and stoichiometric H_2_O) are derived from the cumulative gamma spectra of the two mapping periods from 8 June 2002 to 2 April 2005 and 30 April 2005 to 22 March 2006. The regional extent is limited to the mid-to-low latitudes, since the gamma spectral peaks corresponding to most maps are derived from neutron–nuclei interactions in the Martian regolith, while the underlying neutron spectra are affected by the sharp increase in H abundance at higher latitudes^41^. For consistency with recent work, we use the latest forward-model-derived chemical maps from the gamma spectral mapping periods. This also corresponds to the most rigorous chemical maps available to date. Despite the difficulty of deriving S due to the relative placement of its neutron capture-based peaks in the overall gamma spectral continuum, the resulting maps offer sufficient spatial resolution for analytical work at 5° × 5° to 10° × 10° “pixel” sizes.s Detailed descriptions regarding the fundamentals of deriving elemental wt% from gamma spectra and additional data reduction information can be found in previous works^41,57^. We exclude Al from the comparison due to its coarser resolution by about ×3 compared to the other chemical maps. We also exclude Th given the lack of reported values for in situ chemistry. Mg is excluded by its derivation from mass-balance for chemical maps; non-spectral computation of Na, Ti, Ni, and Mn (e.g. refs.^62–64^) motivated us to exclude them.

To assess the significance of the S and Cl enrichment at the MFF, we used an enhanced Student’s *t*-test^51^, *t*_*i*_, that measures the deviation for each element from the bulk-average of Mars:

1 $$t_i = \frac{{c_i - m}}{{\sqrt {s_{m,i}^2 + s^2} }}$$

where *c*_*i*_ is the mass fraction of Cl (or S), *m* is the global arithmetic mean mass fraction, *s*_*m,i*_ is the numerical uncertainty of *c*_*i*_, and *s* is the standard deviation of the data. The *t*_*i*_ values were calculated for all the elements observed by GRS, but we only included Cl and S results here since we did not observe notable enrichment of other elements at the MFF. We computed the moles of S, *n*(S), and of Cl, *n*(Cl) at each 5° × 5° bin using standard atomic mass values. This takes the form *n*(S) = *w*(S) × 0.03 and *n*(Cl) = *w*(Cl) × 0.029.

### Volumetric calculation of dust on Mars

Radar data indicate the volume fraction of dust in the NPLDs^65^ and the SPLDs to be ~5% and 10%, respectively^65,66^. Given the volume of NPLD and SPLD of 8.21 × 10^5^ and 1.6 × 10^6^ km^3^, respectively^66,67^, if all the dust trapped in the Martian poles were to be distributed globally, it would only make a ~1.4 m thick layer of dust. In Arabia, a dust thickness of more than 20 m has been proposed based on the lack of small craters^68^. Given Arabia’s area of 6.5 × 10^6^ km^2^, the dust, if distributed globally, would make a ~0.9 m layer of dust. The thermal emission spectrometer (TES) on Mars Global Surveyor (MGS) has provided high-resolution maps of thermal inertia (TI) for 85% of the Martian surface^69^, which indicate 19% of the mapped surface of Mars has TI unit consistent with bright unconsolidated fines^69^. Although the thickness of the Martian dust is not known in these regions, HiRISE images indicate dust thickness of at least 4 m near Tharsis^70^. Excluding Arabia and assuming a dust thickness of 5 m, we estimate the total volume of dust to be 84,500 km^3^ in the low-TI regions of Mars. If distributed globally, the dust trapped in the low-TI regions would make a ~0.6 m layer of dust.

### Data availability

All original data described in this paper are archived by NASA’s Planetary Data System.

## Electronic supplementary material

Supplementary Information
