# Supplementary material for: The Medusae Fossae Formation as the single largest source of dust on Mars
Source: Nat Commun. 2018 Jul 20;9:2867. doi: 10.1038/s41467-018-05291-5 (PMC6054634; doi:10.1038/s41467-018-05291-5)
Supplement: Supplementary file 1 — Supplementary Information [file 41467_2018_5291_MOESM1_ESM.pdf]

SUPPLEMENTARY INFORMATION

The Medusae Fossae Formation as the Single Largest Source of Dust on Mars

Ojha et al

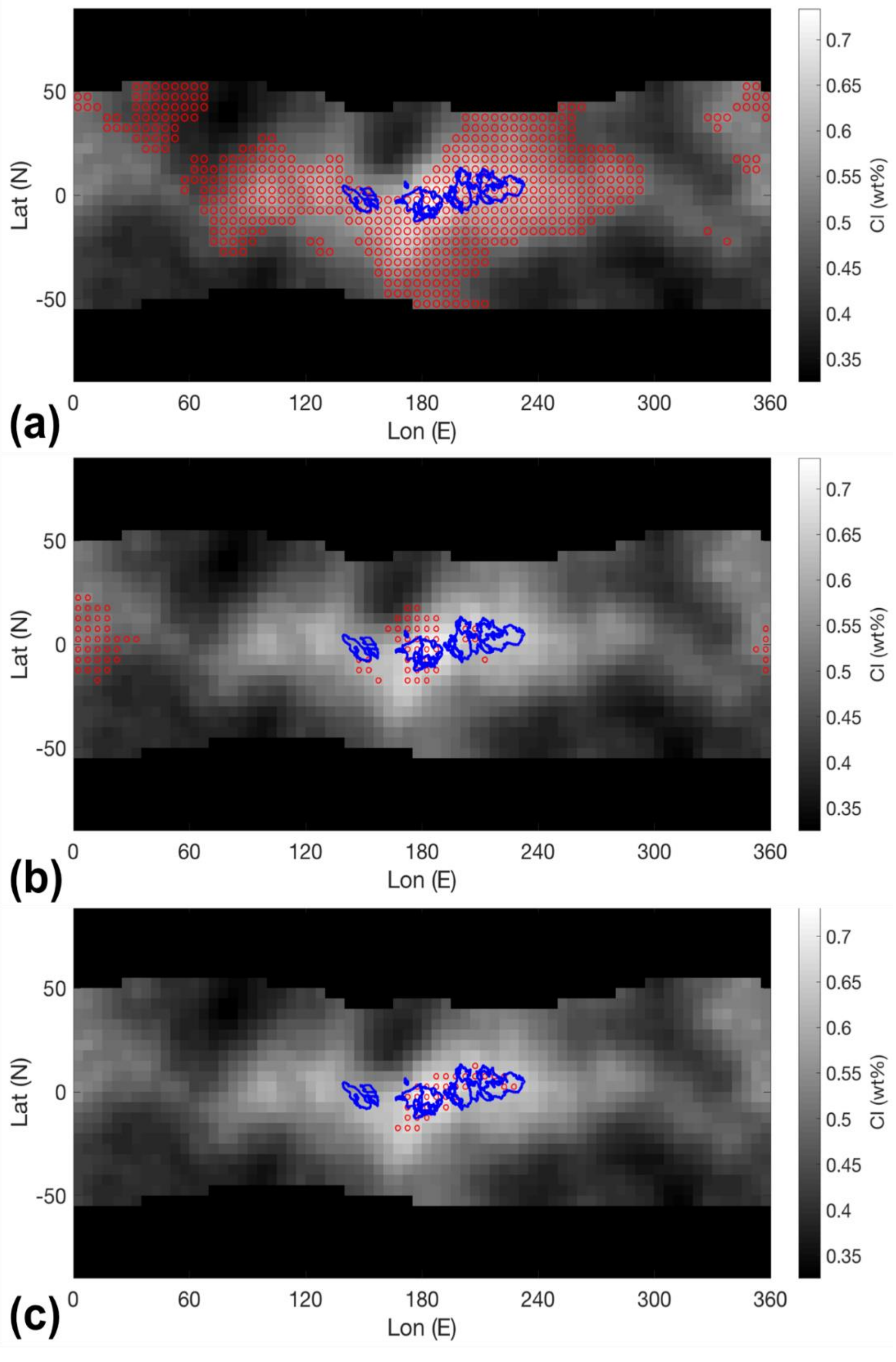

Supplementary Figure 1. Global GRS map showing regions on Mars with enrichment of S, Cl, and S:Cl molar ratio similar to dust, overlain on the Cl wt% map for reference. **(a)** Regions on Mars that have S:Cl ratio between 3 to 4.4 have red empty circles. **(b)** Out of those areas shown in (a), areas with S enrichment as high as that observed in the MFF. **(c)** Out of those areas shown in (b), areas with Cl enrichment as high as that observed in the MFF. The blue outline shows the areal extent of the MFF.

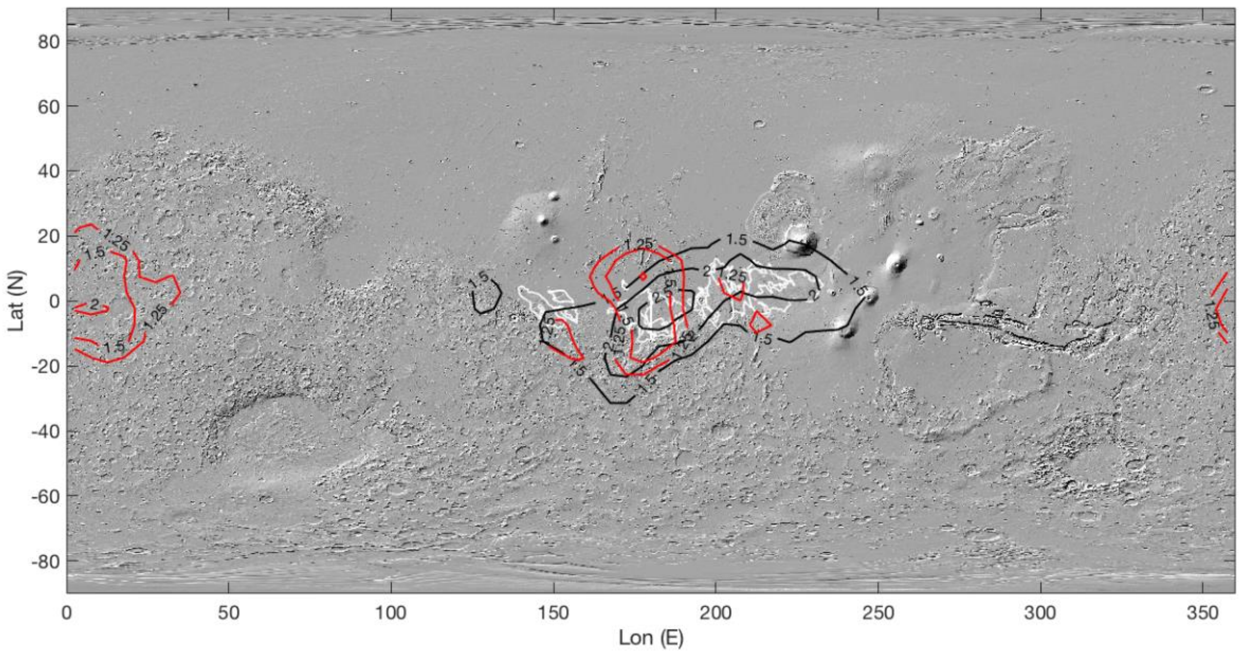

Supplementary Figure 2. Contour map showing regions on Mars with significant enrichment of Cl (in black) and S (in red) based on GRS data. The contour labels corresponds to a modified 't' parameter[8] that show regions with significant enrichment of Cl and S compared to the bulk-average of Mars. The white lines show the areal extent of the MFF.

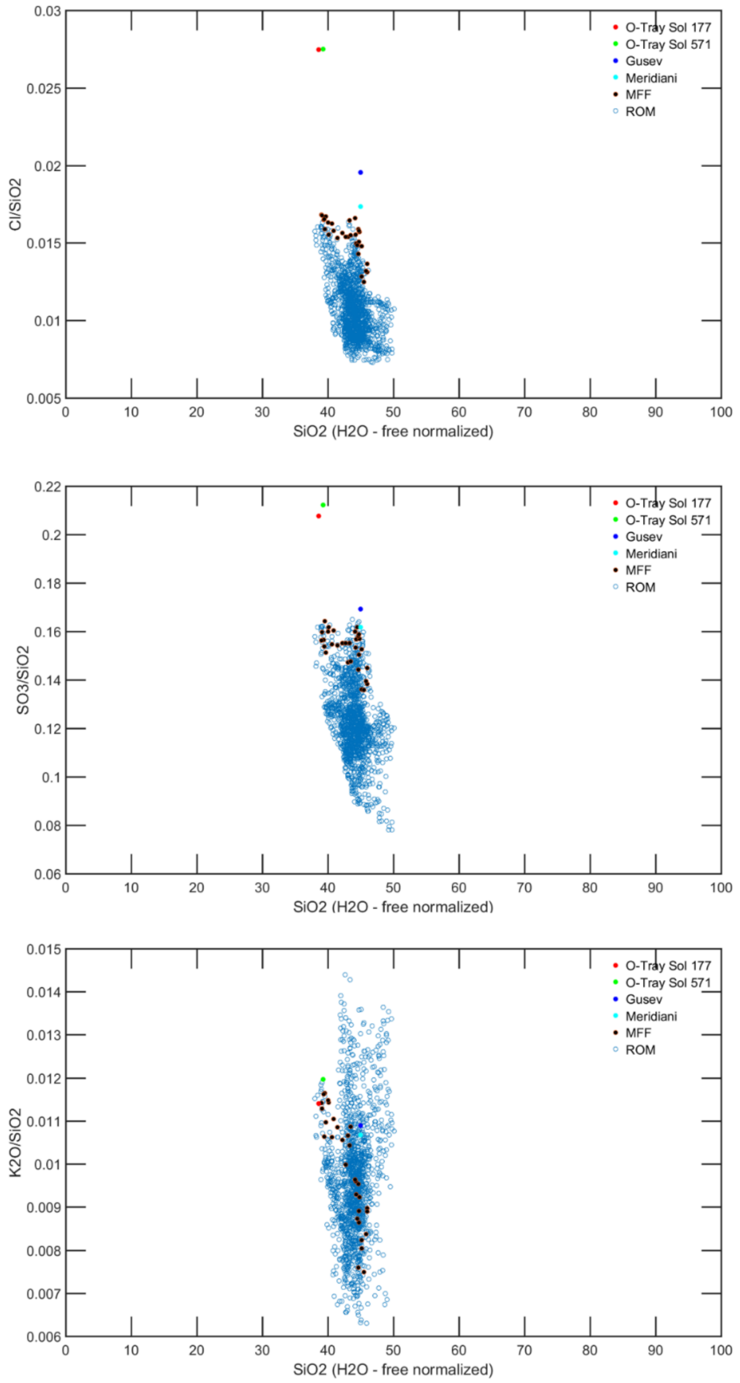

Supplementary Figure 3. Scatter plot of various elemental mass ratio derived from the GRS observations for the entire planet excluding the MFF, denoted rest of Mars (ROM), and in situ dust data. The elemental ratios are plotted against H_2O free normalized SiO2. The normalized factors was calculated for each pixel by using the standard equation[1,2] 100/(100-[H2O]).

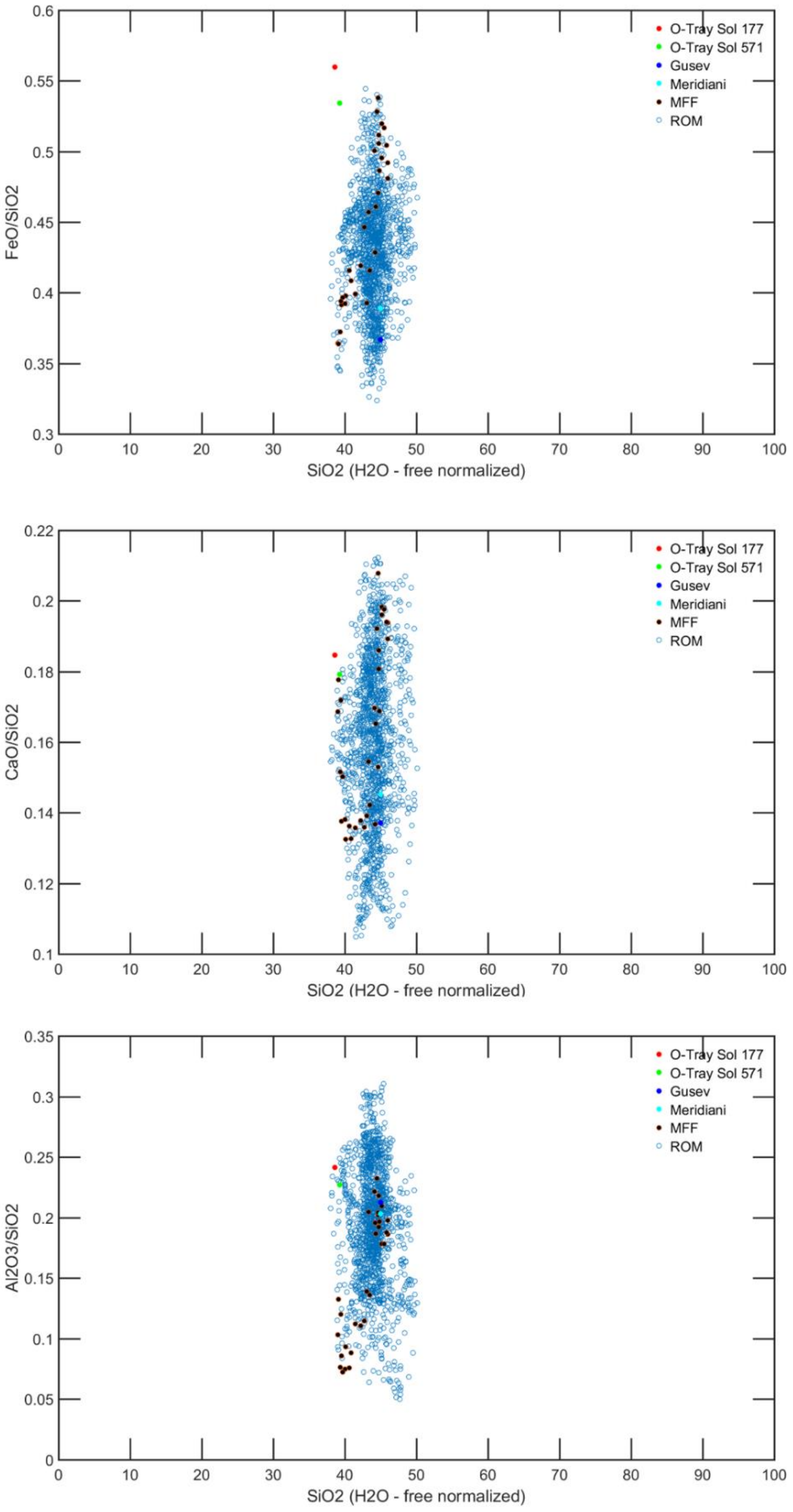

Supplementary Figure 4. Same as Supplementary Figure 3, but for Al_2O_3, CaO and FeO.

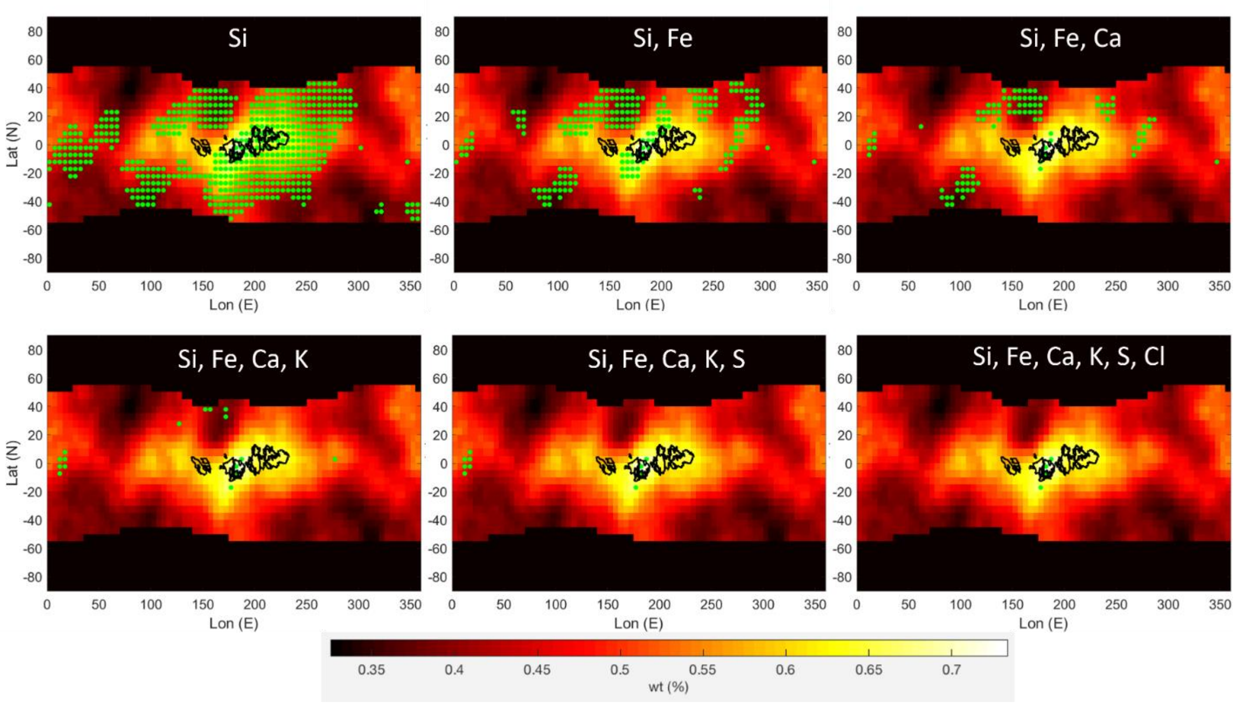

Supplementary Figure 5. Visualization of progressively mapping regions on Mars with similar normalized elemental abundance as that observed for the Martian dust. The green dots show regions of Mars where the normalized abundance of mineral(s) match those observed for the Martian dust. The background map shows Cl enrichment on Mars. The black lines show the outline of the MFF. The only region on Mars where the normalized Si, Fe, Ca, K, S, and Cl abundance collectively match the in situ dust signature is located in the MFF and surrounding area.

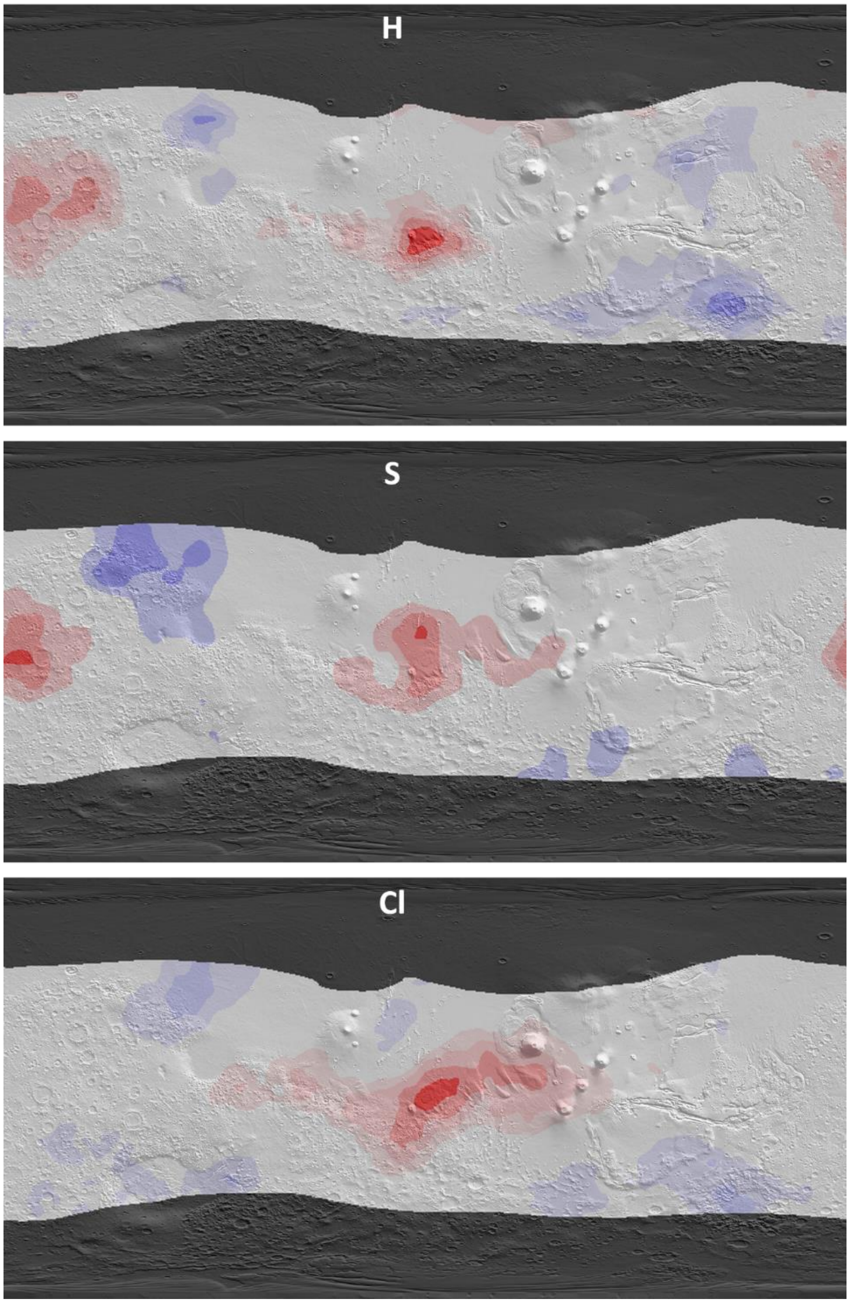

Supplementary Figure 6. GRS derived enrichment-depletion maps for H_2O, S, and Cl. Deepening (light, moderate, and intense) red hues show where the concentration of each element increases at decimeter depths compared to the crustal average in the mid-to-low latitudes. Blue hues show decreasing abundance with depth. The three hue intensities correspond to three thresholds of our enhanced Student's t-test parameter at 68%, 87%, and 95% statistical confidence on a pixel basis.

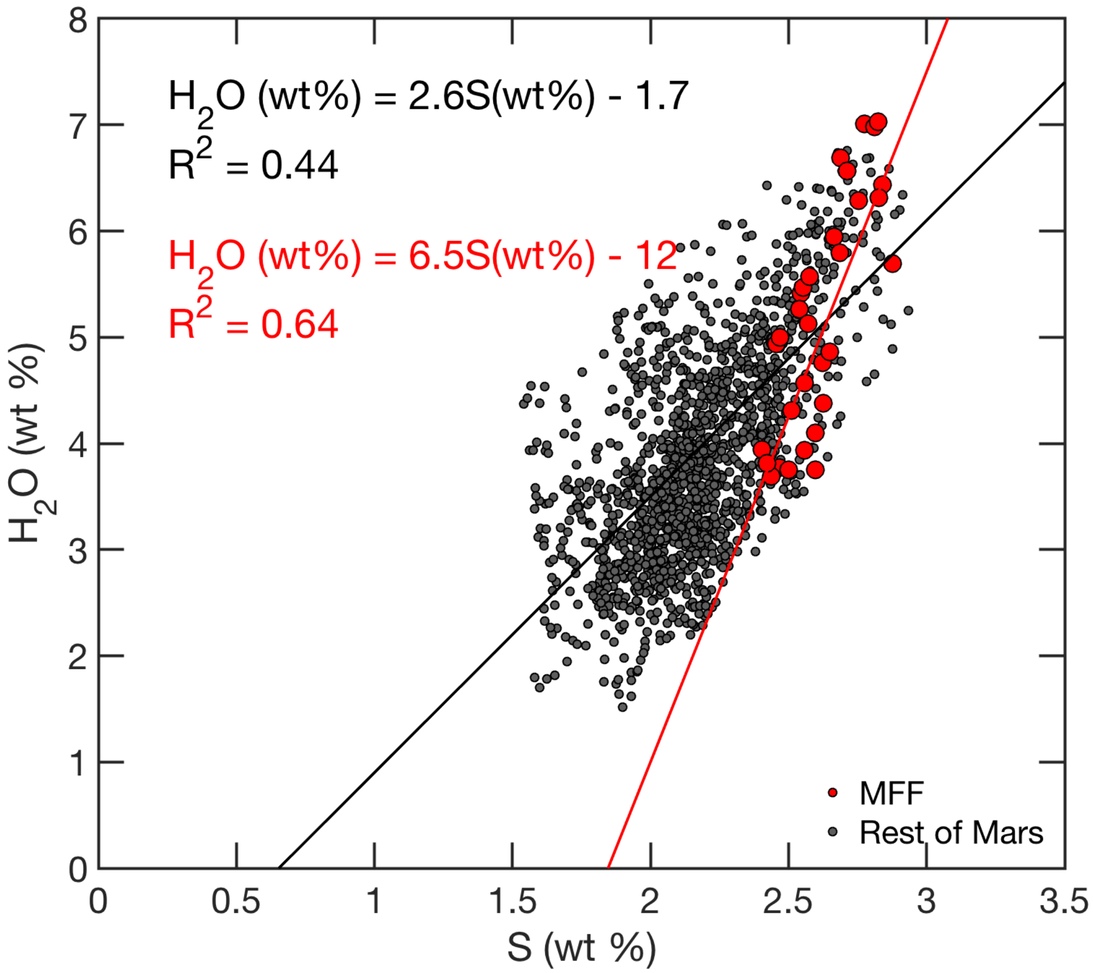

Supplementary Figure 7. Correlation between GRS derived H_2O and S for the rest of Mars (in black) and the MFF (in red). The red line shows the regression for H_2O and S in the MFF. The correlation coefficient, which is simply the square root of R^2 is 0.80 for the MFF. Note the highly negative intercept of the MFF regression line. The black lines shows the regression for H_2O and S for the rest of Mars.

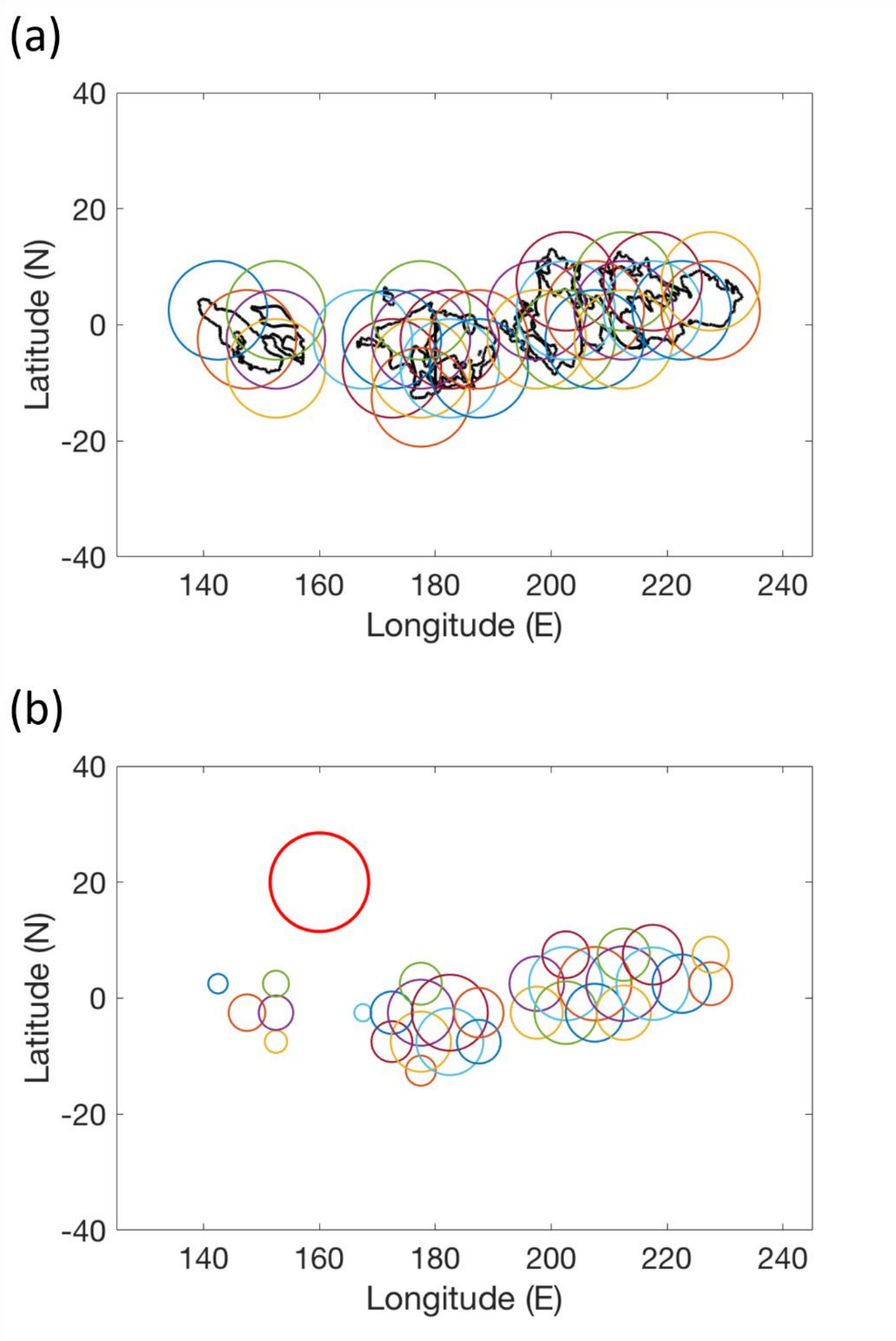

Supplementary Figure 8. (a) Windows of 8.5° radius centered over the MFF. As an instrument collimated only by the atmosphere, the diameter of GRS's intrinsic spatial resolution (i.e., footprint) can be represented by the nadir-centered spherical cap from which 50% of the cumulative spectrum is sourced. While variable with the gamma-ray energy[3], this averages ~4°of arc radius (about 215 - 270 km radius at the equator). If broadened to consider the area within which more than 99% of the cumulative spectrum arises, the effective footprint increases to 8.5° of nadir[4] (as shown in (a)). **(b)** Contribution from the MFF in each of the 32 windows shown in (a). The radius of each circle is scaled to represent the contribution from the MFF. For example, a window with 100% contribution from the MFF would have the same radius as show in ((a), or

the red circle for comparison). Given the relatively small areal extent of the MFF (black outlines in a), GRS observations even within the geometric center of MFF may have substantial external contributions, with only ~77% of the cumulative spectrum attributable to the MFF. The relatively coarse resolution of GRS and limited contribution from the MFF to GRS field of view (a maximum of ~77%) causes a spatial averaging effect, which can subdue the compositional contrast between MFF and the surrounding regions. With the neighboring regions approximating the Martian crustal average chemistry, this can result in lower reported concentrations for Cl and S than in situ reported concentration for Cl and S.

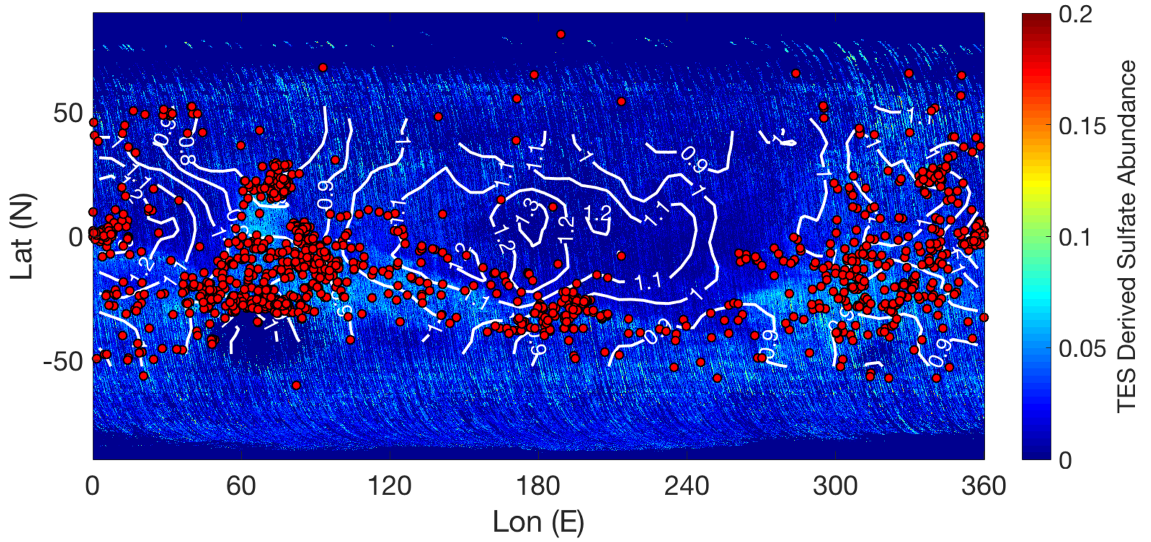

Supplementary Figure 9. Map showing spatial relationships between sulfates detected by orbital spectrometers and elemental S. The background is the TES surface abundance map of sulfates at 4 pixels per degree[5]. Red dots are the locations where hydrous minerals have been detected by (Compact Reconnaissance Imaging Spectrometer for Mars) CRISM and OMEGA. Many of the hydrous minerals detected near Oxia Planus have been sulfates[6,7]. The contours show the relative enrichment of GRS derived S on Mars.

Supplementary References

1. Taylor, G. J., Martel, L. M. V., Karunatillake, S., Gasnault, O. & Boynton, W. V. Mapping Mars geochemically. *Geology* **38,** 183–186 (2010).

2. Susko, D. *et al.* A record of igneous evolution in Elysium, a major martian volcanic province. *Sci. Rep.* **7,** 43177 (2017).

3. Boynton, W. V. *et al.* The Mars Odyssey Gamma-Ray Spectrometer Instrument Suite. *Space Sci. Rev.* **110,** 37–83 (2004).

4. Evans, L. G., Reedy, R. C., Starr, R. D., Kerry, K. E. & Boynton, W. V. Analysis of gamma ray spectra measured by Mars Odyssey. *J. Geophys. Res. E Planets* **112,** (2007).

5. Bandfield, J. L. Global mineral distributions on Mars. *J. Geophys. Res.* **107,** 5042 (2002).

6. Ehlmann, B. L. & Edwards, C. S. Mineralogy of the Martian Surface. *Annu. Rev. Earth Planet. Sci* **42,** 291–315 (2014).

7. Carter, J., Poulet, F., Bibring, J. P., Mangold, N. & Murchie, S. Hydrous minerals on Mars as seen by the CRISM and OMEGA imaging spectrometers: Updated global view. *J. Geophys. Res. E Planets* **118,** 831–858 (2013).

8. Karunatillake, S. *et al.* Chemically striking regions on Mars and Stealth revisited. *J. Geophys. Res. E Planets* **114,** (2009).
